# Opponent or allied? An European analysis of the union presence and human resource practices

**DOI:** 10.3389/fpsyg.2022.878006

**Published:** 2022-07-25

**Authors:** Inés Martínez-Corts, Juan Pablo Moreno-Beltrán, Santiago Renedo, Francisco J. Medina

**Affiliations:** Department of Social Psychology, University of Seville, Seville, Spain

**Keywords:** human resource policies, unions, wellbeing, trust, justice

## Abstract

Human Resources Practices (HRPs) and unions coexist in some organisations to manage the employment relationships of the workers. In this study, we analyse how the presence/absence of unions and HRPs are combined in private European organisations, and which of these combinations are related to higher levels of wellbeing and the quality of labor relations. Data come from 24,503 workers of private organisations, obtained from the Sixth European Working Conditions Survey. Latent profiles analysis and different analyses of the variance suggested four different profiles. The profile with the greatest presence of HRPs and union presence is related to the highest levels of employees' wellbeing and quality of labor relations in organisations, whereas those organisations with a low level of union presence or HRPs reached the worst levels in employees' wellbeing and quality of labor relations indicators. The results and their practical implications are discussed.

## Introduction

The improvement of working conditions and work–life quality for employees has traditionally been accomplished in two different ways: collective bargaining - through pressure tactics applied by the employees' representatives, and through the Human Resource Practices (HRPs) implemented by organisations. Collective bargaining refers to the negotiation practice legally established between the employees and the representatives, where they agree on their employment conditions, mainly salary, working hours, and holidays (Elgoibar et al., 2016). On the other hand, the HRPs refer to the organisational practices that seek to improve workers' competencies and organisational performance (Beijer et al., 2019). This dichotomy has been the base of labor relations for the last 70 years, combining both collective bargaining – through the representatives' influence – and the HRPs led by the organisation.

During the last decades, employees' representativeness has reduced power in western organisations, both in Europe and in the United States (Verma et al., 2002; Ibsen and Tapia, 2017; Koçer, 2018). This loss of power has two main causes: a decline in the level of trade union membership across Europe (loss of associational power) and some changes in the laws that regulate collective bargaining that has been gradually unbalanced toward the employer side (loss of institutional power). These two facts have reduced the trade unions' influence in the industrial relations setting. Even so, strikes and tensions between workers and employers and even with governments worldwide have increased for years, even in the new economy businesses, as we saw in 2021 around the world with delivery drivers, due to their bad employment conditions (Feliz-Leon, 2021).

Some inquiries have tried to explain the emergence of HRPs in sectors almost previously non-existent, such as small and medium-sized companies, as a consequence of the reduction of the power of the representatives as if the relationship between the two was a zero-sum matter (i.e., Machin and Wood, 2005). In this sense, Rogers and Freeman (2006) argue that the representatives may constitute a hindrance to the implementation of the HRPs as if the presence of one inhibited the other or *vice versa*. However, other authors consider that the increase of HRPs in organisations and the decrease of representatives are not related, the emergence of HRPs is due to a strategic decision by organisations to increase employees' productivity and commitment (Guest and Conway, 1999). Kochan and Osterman (1994) even claim that the representatives could facilitate and enhance the new labor relations where the representative power coexists with the HRPs. In summary, the evidence on how representatives and HPRs coexist in organisations is contradictory.

Guest and Conway (1999) tried to analyse this dichotomy according to the type of organisation where the HRPs and representatives were present. They found that HRPs and unions coexisted only in an emergent sector called the New Deal, with a majority of start-up organisations. There were also some organisations without any existing HRPs or employee representatives, called “Black Holes.” Organisations from the British public sector (where collective labor relations prevailed and there were hardly any HRPs) and industrial organisations (where HRPs prevailed and there was no collective bargaining) were not integrated into this study.

In the last 20 years, organisations have changed, with further diversification specifically in the private sector. Diversification is related to aspects such as globalization of the business, the proliferation of new organisational and contractual categories, such as temporary employment companies (TEA), the self-employed who perform stable tasks in the organisation such as transportation and distribution, outsourcing of parts of the business, or self-employment that provides temporary services in the Gig Economy sector. HRPs have extended to sectors where they were previously non-existent, such as NGOs (Cabrera et al., 2020) or small organisations (Bryson and White, 2018). There has also been an increase in technological organisations, or professionalization both in the service and production sectors.

At the same time, in the last three decades, the change in the labor relations seems to be translating into a decrease in the presence of representatives in most Western countries (Schnabel, 2013). Specifically, in the countries of the European Union, the rate of union membership and the number of unions has progressively decreased from the 2000s until today (Visser, 2019). This reduction in union presence is drawing a new profile in labor relations in the new business environment. Greater individualization and greater power of organisations are being favored throughout the labor relations policies. So, the current tendency seems to be to transfer issues such as work enrichment or changes in working conditions to the individual negotiation, including new issues such as job crafting or idiosyncratic deals in the negotiation agenda (Martinez-Corts and Demerouti, 2017; Euwema et al., 2019; Martínez-Corts and Moreno-Beltrán, 2020). In short, the economic and organisational context seems very different from the one at the end of the last century, so the systematic study of the new private organisational environment is of interest.

In this new economic and organisational context, it is particularly relevant to analyse the presence or not of HRPs and unions and how they interact, as both have an impact on employee wellbeing and/or the quality of labor relations. The term quality of labor relations is defined as the quality of the dialogue that employees have with companies (Addison and Teixeira, 2019). In that sense, HRPs enable the attraction and retention of specialized talent (Ng et al., 2010), which is highly in demand in organisations with a high technological level, and favor a trust climate (Cristiani and Peiro, 2015), employees' engagement, and organisational commitment (LaTorre et al., 2016). However, the effect of the union presence is not so clear. Although union influence has made possible to protect and advance workers' rights and interests, it is also proved to be dysfunctional in some contexts, in the face of processes of change, or in very diverse organisations where groups of workers may feel that union demands do not address their specific concerns. This justifies that while some studies have found that union presence can have a strong impact on organisational performance in an organisational change context (Butler and Tregaskis, 2018), or increase employees' perceptions of distributive justice (Bryson et al., 2013) and organisational trust (Newman et al., 2019), it has also been demonstrated that these effects can be diminished in a conflict climate or when there are few resources to achieve union's proposed changes (Gregory and Milner, 2009). Therefore, it is of interest to analyse the effects of not only the presence of HRPs or unions independently but also the effect generated when both are combined.

Therefore, the current study aims to update the Guest and Conway study by doing a similar study 15 years later on how organisations are distributed according to how they combine HRPs and union presence. However, we go further by involving any type of private organisation and extending the research to include organisation in all European countries. To do this, we used data provided by the Sixth European Working Conditions Survey, with 24,503 workers of European private sector organisations. Second, we analyzed the outcomes of these organisational profiles in terms of employees' wellbeing and the perceived quality of their labor relations. This study contributes to the literature by showing evidence of the relationship between HRPs and union presence with employees' wellbeing. First, as far as we know this is the first study that describes how union presence may relate to employees' wellbeing. Second, we expand the person-center HRPs studies by analyzing how the combination of the two main sources of personnel policies (trade union influence and human resources departments) have an impact on employees' wellbeing. At a practical level, the results of this study will contribute to the understanding of how employers encourage employee participation through union representation while developing the human resources department's understanding of how cooperation between the two will benefit not only the employees but also the organisation as a whole

## Theoretical framework

### The role of human resource practices in organisations

One of the most accepted definitions in the literature describes HRPs as the set of strategies, policies, and practices developed by the company to influence employees' behavior, performance, and attitude to achieve the organisational goals (Delery and Doty, 1996). According to the objectives pursued, the HRPs can be classified as focused on the performance — that is, performance-centered or on the employee – that is, person-centered HRPs. The objective of the former is to increase the performance of workers in the short term. It has been traditionally named hard practices because they emphasize the “resource” aspect of the employees to improve organisational performance (Legge, 1995). Some of these practices imply the implementation of a reward system or performance evaluation. Additionally, person-centered HRPs or soft practices emphasize the human aspects of management highlighting the potential of employee skills development, motivation, self-fulfillment, and contentment with a meaningful job to enhance the competitive advantage of organisations in the long-term (Cristiani and Peiro, 2015). Specifically, person-centered HRPs have the potential to attract and retain highly qualified employees by developing employee skills, counting on them for decision making, and offering participation practices (Ng et al., 2010). In this study we analyzed job enrichment – that is, the degree to which organisations promote work task variety, new learnings, and the use of a variety of skills (Hackman and Oldham, 1976; Govender and Parumasur, 2010), participation at work, that is, the degree to which organisations encourage employees' participation in work decision making, employees' voice and the possibility to suggest work improvements (Chen and Huang, 2009; Bücker and Van Der Horst, 2017); and training and development programmes that are intended to develop employees' skills, abilities, and competencies (Takeuchi et al., 2007). As person-centered HRPs, all these HRPs aim to develop employees' commitment, and focus on a win–win employee–organisation long-term relationship.

As demonstrated by Cristiani and Peiro (2015) and Conway et al. (2016), these practices have a positive impact on employees' wellbeing, and consequently on productivity. Moreover, person-centered HRPs are of relevance in organisations since they contribute to enhancing the organisational competitive advantage. Specifically, they have the potential to attract and retain highly qualified employees. For instance, organisations that promote the development of employee skills, count on them for decision making, and offer participation practices, will have a much higher probability of attracting and retaining talent (Ng et al., 2010). These practices have an impact on the aspects that link the person to the organisation and on the processes that promote identification with the organisation (Cristiani and Peiro, 2015). These arguments justify that in our study, person-centered HRPs, that is, participation at work, training, and job enrichment, are considered. As person-centered HRPs, all these HRPs aim to develop employees' commitment, and focus on a win–win employee–organisation long-term relationship.

### The role of unions in organisations

Unions are one of the pillars of labor relations in democratic countries. Their purpose is to assure the voice and participation of employees as well as the protection of their interests and rights, improving their working conditions. According to the classical studies of (Webb and Webb, 1920), unions are defined as the continuous association of employees to maintain or improve the conditions of their working life. The presence of unions throughout the 20th century has prompted improvements in working conditions, going from semi-slavery at the beginning of the century to the current conditions in which labor rights are generally guaranteed in most countries of the world.

Unions might influence through several mechanisms, first by using formal and institutional channels to transfer the voice from employees to the company, second through facilitation processes from organisation to employees conveying employers' practices to workers and also from employees to organisation, supporting employees in internal processes such as conflicts, harassment, or grievances (Bacon and Hoque, 2012).

The intervention of unions has not only provided continuous improvements in employee working conditions throughout history, but it has also had a positive impact on organisational productivity Freeman and Medoff (1984). For example, Butler and Tregaskis (2018) illustrated how collaboration between unions and the organisation allows for the increase of the capability of the organisation to overcome complex change situations through the effect of the collective voice. Moreover, in situations of organisational change, unions contribute to maintaining higher levels of distributive justice (Bryson et al., 2013). Other studies conclude about positive organisational outcomes when a good climate between the management and the unions exists (Valizade et al., 2016). For example, Newman et al. (2019) found that, in a cooperative organisational climate, unions have a positive effect on organisational trust. Therefore, the quality of labor relations may be reinforced by union labor.

However, some authors consider that unions can also have a negative impact not only on organisations but on employees. When there is a conflict climate, when unions are reluctant to relevant organisational changes, or when certain agreements are very costly to implement, the influence of unions could be negative for organisations, increasing dysfunctional conflicts and affecting the organisational development (Gregory and Milner, 2009).

Trade union influence can also be a barrier to improvements in employees' working conditions if their bargaining agenda does not match the issues relevant to some sectors of the workforce. Collective bargaining is traditionally focused on distributive issues, such as pay, working hours, or holidays. Other issues that may be relevant to the wellbeing of employees such as flexibility, work-life balance, or health promotion are not usually part of collective bargaining agendas (Elgoibar et al., 2021). In this sense, and related to specific female concerns, some researchers have argued that the collective bargaining agenda has a strong gender bias and issues of relevance to women are rarely addressed (Gregory and Milner, 2009). For that, recent evidence demonstrates, in a case study, that trade union presence and collective bargaining are ineffective in producing changes in the flexibilization of work and job quality improvement (Wood, 2016). For this reason, the analysis of the contexts in which union influence is effective is of interest to labor relations research.

### The combination of HRPs and union presence

Up until the early 1990s, union influence in politics and organisational dynamics was limited to specific sectors and specific organisational sizes. In public sector organisations, the union had the power to influence the organisational practices, leaving the human resources departments as mere units of personnel administration. In the private sector and large organisations, it was the human resources departments that set the practices that affected workers. The small and medium-sized company sector, which constitutes the majority in Europe, had neither aspect: neither union influence nor HRPs. Finally, the 1990s was a decade with an enormous social and organisational disruption with the expansion of the tech industry, when the two aspects coincided simultaneously. This typology generated according to the presence of HRPs and union participation (UP) referred to in previous paragraphs (Guest and Conway, 1999) generated four types of organisations: “The New Realism” (high HR/high UP), “Individualized HRM” (high HR/low UP), “Traditional Collectivism” (low HR/high UP), and “The Black Hole” (low HR/low UP). The sample for this study was drawn from the 1996 British National Employment Survey, made up of 1,000 British workers mostly from the private sector.

After this seminal article, Verma's meta-analysis in 2005 explained how unions could favor or hinder the implementation of HRPs in organisations. He reached contradictory results. First, this author found that unions could facilitate the implementation of HRPs. For example, if unions are present in the direction of training programmes, the receptivity of employees to such programmes increases. Also, unionized organisations report more voice and participation mechanisms, which increases the quality of decision making. Recently, Cristiani and Peiró (2018) and (Yang and Tsou, 2018) found similar facilitation outcomes in China and Uruguay, respectively. Second, unions can have negative effects on the implementation of HRPs, namely, wage flexibility. As suggested by Verma (2005), unions are adamantly opposed to incentive programmes, variable pay, and flexible work practices, considering their practices that favor the organisation and are not employee oriented. Finally, the results of the study conducted by Verma (2005) show how unions would not have a significant impact on other HRPs such as job rotation or innovation practices.

One factor that could explain the contradictory results is the regulatory context in each country. While some studies show negative effects on productivity in European countries such as France or the United Kingdom (Bryson et al., 2011), meta-analytical studies find contradictory effects, for example, positive effects are found in the United States and negative effects are found in various European countries (Doucouliagos and Laroche, 2003). In this study, we aim to shed light on the explanation of these inconsistent effects. We also go beyond Guest and Conway (1999) study because their research was focused only on the United Kingdom, and the present study analyzed participants from different European countries. Drawing on Guest and Conway's (1999) patterns, the interest of the current research is to identify different types of organisations according to the specific combination of HRPs and union representation. Furthermore, we explored individual-centered HRPs because they have a greater capacity to generate wellbeing and improve the quality of labor relations. Therefore, we predict that:

**Hypothesis 1**. Four profiles, based on the presence and the absence of HRPs and trade union representatives, will be identified.

### Effects of different combinations of human resources practices with union influence

Both the employees' wellbeing and the perception of the quality of labor relations are relevant outcomes since they have an impact on organisational performance (Albrecht et al., 2015; Ambrose et al., 2019). The signaling theory explains how HRPs are perceived by the employee as a symbol of support, care, and improvement of their working conditions by management (Connelly et al., 2011). Thus, having the belief that the organisation cares about their needs, the employees, through a cognitive consistency mechanism (Festinger, 1957) could adopt attitudes that are coherent with these feelings, improving engagement and organisational commitment (LaTorre et al., 2016). For example, if an employee can participate in decision-making processes, he/she can acquire a prosocial motivation toward the organisation (Grant and Ashford, 2008). Prosociality generates trust within the organisation, with coworkers, and with supervision (McElroy, 2001; Morrison, 2014). Moreover, participation can contribute to employees' perception of organisational transparency, increasing their perception of justice (Bowen and Ostroff, 2004). These mechanisms (i.e., motivation and justice) have a significant effect on employees' wellbeing. Besides, as explained by the self-determination theory (Ryan and Deci, 2000), enrichment and training practices have a positive impact on employees' wellbeing and the quality of labor relations because they allow them to flourish and achieve the proposed objectives, beyond having the opportunity to face new challenges and progress (Tremblay et al., 2010). Opposite, the lack of HRPs can be interpreted by the employee as a lack of support from the organisation for their needs, enhancing their desire to leave (Slavich et al., 2014).

Contrary to the HRPs, the role of unions has barely been analyzed in the promotion of employees' wellbeing. Most of the studies have mainly been focused on analyzing the effects of union presence on productivity and organisational profits (Doucouliagos and Laroche, 2009). However, having mechanisms to participate in the organisation, as in the case of unions, can also have a positive impact on employees' wellbeing, because it allows them to express their voices and concerns (Reissner and Pagan, 2013; Yang et al., 2019). In fact, one of the purposes of unions is to promote a collective voice with employees' requirements and negotiate those requirements.

Beyond the effect of HRPs and union presence solely the combined effect of the human resources departments and unions should be analyzed. For this combination to be effective, synergies must exist between the people responsible for designing and implementing the HRPs, for example, managers, human resources departments, and representatives. For instance, representatives may shape how employees perceive the implementation of HRPs, promoting cooperation or conflict among employees and managers (Bowen and Ostroff, 2004). The collaboration between representatives and managers or HR departments has become increasingly intense, especially since the crisis of 2008, where flexibility practices were necessary to guarantee the survival of many companies. In this sense, HRPs implementation and union presence are not mutually exclusive. Some studies suggest a new perspective called “Mutual Gains.” For instance, Cristiani and Peiro (2015), in the Uruguayan context, showed that union presence is positively related to person-centered HRPs. Moreover, Pohler and Luchak (2015), in the Canadian context, found that the collaboration between business management and unions was the key to minimizing the possible negative and enhancing the positive effects on organisational outcomes of union presence. When the union verifies that HRPs are aligned with and employee-focused organisation strategy, for example, invest in employment as a competitive advantage implementing person-centered HRPs, they interpret the relationship as co-operative, enhancing the union's reciprocate cooperation (Eisenberger et al., 2001). In turn, Yang et al. (2019), in the Chinese context, found that unions and HRPs work synergistically, having positive effects on the quality of relationships between social agents, and improving employees' engagement.

Based on this previously discussed research, we predict that:

**Hypothesis 2**. The four combinations of the presence and the absence of HRPs and trade unions will differentially relate to employees' wellbeing and the quality of labor relations in the following way:

**Hypothesis 2a**. The presence of HRPs will have a positive relationship with employees' wellbeing and the quality of labor relations.

**Hypothesis 2b**. The absence of HRPs and trade unions will decrease the employees' wellbeing and the quality of labor relations.

**Hypothesis 2c**. The combination of HRP and trade union will have a positive relationship on employees' wellbeing and the quality of labor relations more than HRP or union alone.

## Method

### Procedure and participants

The data were collected from the sixth European Working Conditions Survey (EWCS). The sixth EWCS is a face-to-face survey that covers the 28 E.U. Member States. In the current study, we focus on the private sector data set (*N* = 24.503). On average, 875 employees participated per country, ranging from 453 (i.e., Montenegro) to 2,038 (i.e., Spain). The percentage of men (55, 23%) was higher than women (44, 74%); the mean age was 41.39 years (SD = 12.39). Participants were representatives from small (2–9 employees; 36, 5%), medium (10–249 employees; 39.3%), and big organisations (more than 250 employees; 24.3%); 78% of the overall participants had a full-time contract and 16 (8%) had a part-time contract. The majority of the participants had a permanent contract (76.6%), 10% had a non-permanent contract, 1.2% had a contract with a temporary employment agency, and 0.6% had an internship contract. The sixth EWCS assessed the following aspects: support, trust, cooperation, wellbeing, employment conditions, employee participation at work, HRPs, and work organisation. The sixth EWCS is revised with the support of multi-disciplinary experts, and it is developed in 49 language versions. Eurofound implements rigorous procedures to accurately obtain the best results possible in different cultures.

### Measures

To analyse the characteristics of the organisations, two indicators were used: activity sector and organisation size.

*Activity sector*. The primary sector comprises those organisations whose main activity is obtaining source materials from the natural environment: livestock, agriculture, fishing, and mining and logging. The secondary sector includes organisations that are destined to transform raw materials: industry and construction. The third sector includes all economic activities that provide services such as transport, commerce, tourism, healthcare, education, and finances.

*Organisation size:* small, medium, and big organisations.

Employees' working contracts were also analyzed as they contribute to define their working conditions. Working contracts were defined based on the working hoursfull-time and part-time – and the contract period (temporal, permanent, …).

*HRPs* were measured with three indicators: participation at work, training, and job enrichment.

*Participation at work*. Participants were required to “Please select the response which best describes your work situation”: “You are consulted before objectives are set for your work,” “You are involved in improving the work organisation or work processes of your department or organisation, “You have a say in the choice of your work colleagues,” and “You are able to apply your own ideas in your work.” A five-point response rate scale was used (1 = never, 5 = always). Cronbach alpha was 0.71.

*Training* was measured with two items following the question: “Over the past 12 months, have you undergone any of the following types of training to improve your skills?”: “Training paid for or provided by your employer” and “On-the-job training (co-workers, supervisors).” All questions were dichotomous, (0 = no; 1 = yes). Cronbach alpha was 0.72.

*Job enrichment*. Variety in work was measured with three items: “Does your job involve rotating tasks between yourself and colleagues? Do those tasks require different skills? and “Generally, does your main paid job involve learning new things?. All questions were dichotomous, (0 = no; 1 = yes). Cronbach alpha was 0.70.

*Union presence* was measured with three items, introduced by: “Does the following exist at your company or organisation?”: “Trade union, works council or a similar committee representing employees,” “Health and safety delegate or committee,” “A regular meeting in which employees can express their views about what is happening.” All questions were dichotomous (0 = no; 1 = yes). Cronbach alpha was 0.94.

Employees' wellbeing was measured with two indicators: work engagement and general wellbeing.

*Work engagement* was measured with five items introduced by: “Please tell me how often you feel this way”: “At my work I feel full of energy,” “I am enthusiastic about my job,” “Time flies when I am working,” “I feel exhausted at the end of the workday” and “I doubt the importance of my work.” The last two items are reverse-coded. A five-point response rate scale was used (1 = never, 5 = always). Cronbach alpha was 0.72.

*General* wellbeing was measured with five items introduced by the question: “Which is the closest to how you have been feeling over the last 2 weeks?”: “I have felt cheerful and in good spirits,” “I have felt calm and relaxed,” “I have felt active and vigorous,” “I woke up feeling fresh and rested,” and “My daily life has been filled with things that interest me.” A five-point response rate scale was used (1 = never, 5 = always). Cronbach alpha was 0.75.

The quality of labor relations was measured with three indicators: employees' justice perception, perceived social support, and leadership quality.

*Employees'* j*ustice perception* was measured with three items introduced by “To what extent do you agree or disagree with the following statements?”: “Conflicts are resolved in a fair way”, “The work is distributed fairly” and “You are treated fairly at your workplace.” A five-point response rate scale was used (1 = strongly disagree, 5 = strongly agree). Cronbach alpha was 0.75.

*Perceived social support* was measured with two items followed by the sentence: “Please select the response which best describes your work situation”: “Your colleagues help and support you” and “Your manager helps and supports you.” A five-point response rate scale was used (1 = never, 5 = always). Cronbach alpha was 0.72.

*Leadership quality* was measured with six items following the question: “To what extent do you agree or disagree with the following statements?”: “Your supervisor respects you as a person,” Your supervisor gives you praise and recognition when you do a good job, “Your supervisor is successful in getting people to work together,” Your supervisor is helpful in getting the job done,” “Your supervisor provides useful feedback on your work,” “Your supervisor encourages and supports your development.” A five-point response rate scale was used (1 = strongly disagree, 5 = strongly agree). Cronbach alpha was 0.72.

### Analysis strategy

#### Preliminary analysis

To analyse the construct validity and discrimination between the three HRPs, namely, participation at work, job enrichment, and training, and the trade union presence, a confirmatory factor analysis (CFA) was carried out using MPLUS. For this purpose, three fit indices were evaluated: chi-Square (χ^2^/*p*), Root Mean-Square Error for Approximation (RMSEA of < 0.08), and Comparative Fix Index (CFI of > 0.95) and Tucker Lewis Index (TLI of > 95) (Schreiber et al., 2006).

The latent profile analysis of the MPLUS program was used to identify latent profiles based on the survey data. We used the factor scores sourced from the previous confirmatory factor analysis. With continuous variables, MPLUS estimates factor scores by using a regression method and also with categorical variables by using the Maximum A Posteriori (MAP) method (Asparouhov and Muthén, 2010). To determine the correct number of latent profiles, Muthén and Muthén's (2017) guidelines were followed. The Bayesian Information Criterion (BIC), the Bootstrap Likelihood Ratio Test (BLRT), and the entropy were used to check the fit of the latent profiles model.

To analyse the differences between profiles related to dependent variables, namely, perceived justice, social support, and leadership quality, we conducted six univariate analyses of variance (ANOVA) using profiles as a grouping variable. *Post-hoc* pairwise comparisons were carried out using Tukey's *t* test.

## Results

### Descriptive statistics

Table 1 shows descriptive statistics of the main variables of this study. As we can see, all HRPs are positively correlated. Both HRPs and union presence are positively correlated to the quality of relationship and employees' wellbeing.

**Table 1:** Descriptive statistics and correlations between dimensions.

	**Correlations**								
	**1**	**2**	**3**	**4**	**5**	**6**	**7**	**8**	**9**
1. Participation at work	–								
2. Training	0.07^**^	–							
3. Job enrichment	0.19^**^	0.24^**^	–						
4. Trade U. presence	0.14^**^	0.30^**^	0.18^**^	–					
5. Justice	0.34^**^	0.04^**^	0.02^**^	0.07^**^	–				
6. Social support	0.39^**^	0.08^**^	0.21^**^	0.09^**^	0.47^**^	–			
7. Leadership quality	0.41^**^	0.12^**^	0.10^**^	0.11^**^	0.63^**^	0.56^**^	–		
8. Engagement	0.08^**^	0.02^**^	0.01^*^	0.02^**^	0.10^**^	0.07^**^	0.09^**^	–	
9. General wellbeing	0.22^**^	0.05^**^	0.04^**^	0.07^**^	0.42^**^	0.28^**^	0.36^**^	0.12^**^	–
*Mean*	6.02	0.59	1.54	0.66	4.00	3.93	4.94	4.04	4.43
*SD*	11.54	0.76	1.12	0.48	0.84	1.00	0.90	2.92	1.00

* *p < 0.05*.

** *p < 0.01*.

### Confirmatory factor analysis

Before testing our hypotheses, we first assessed the factor structure of our model, including four latent predictor variables: participation at work, job enrichment, training, and union presence. Results of the four-factor model indicated an acceptable model fit, χ^2^ (48, *N* = 24,503) = 3,880.09, *p* < 0.001, RMSEA = 0.057, CFI = 0.921, TLI = 0.891. To assure that the four-factor model did fit the data better than other alternative models, we tested alternative models, such as a three-factor model (unifying two HRPs). The model's fit indices were not satisfactory [χ^2^ (51, *N* = 24,503) = 5,711.92, *p* < 0.001, RMSEA = 0.067, CFI = 0.884 and TLI = 0.850]. When we combined all the three practices to form an overall HRPs factor, the model's fit indices were significantly worse than the four-factor model, χ^2^ (53, *N* = 24,503) = 12,508.81, *p* < 0.001, RMSA = 0.098, CFI = 0.744, TLI = 0.681. Since the fit of such alternative models was less acceptable, the four-factor model (job enrichment, training, participation at work, and trade unions presence) formed the basis for further analyses. The factorial weights were found to be >0.50 (Nunnally and Bernstein, 1994). Following Muthén and Muthén (2017), we used the factor scores from the CFA to run the latent profile analysis.

### Analysis of latent profiles

To determine the proper number of latent profiles, we used different criteria: LogL, Bayesian Information Criterion (BIC), the Bootstrap Likelihood Ratio Test (BLRT), and entropy and posterior probabilities (avePP). According to the literature, the lower the values of LogL, BIC, and BLRT, the better the fit of the model. Entropy values ranging from 0 to 1 indicate the classification error of the model. The closer the value is to 1, the smaller the classification error of the model. Finally, each latent profile should consist of at least 5% of the total number of samples, and the probability of belonging to each profile should be more than 70% (Nagin, 2005).

Five models with one to five latent profiles were used (see Table 2). The values of LogL, BIC, and BLRT gradually decreased from Model 1 to Model 5, the *p* of BLRT was significant at the 0.05 level from Model 1 to Model 4, not being significant in relation to model 5 with model 4. Entropy improved from 0.8 to 0.85 in model 4 and entropy was >0.9. Furthermore, the BLRT *p* for the four-profile model was significant, but not for the five-profile model. Therefore, Model 4 was considered the most suitable. We determined that the four-profile model fit the data best.

**Table 2:** Model fit statistics.

**Model**	**BIC**	**BLRT**	**Entropy**	**LogL**
1-Profile	12,4505.360	–	–	−62,224.965
2-Profiles	94,314.964	29,679.000^*^	0.81	−47,091.789
3-Profiles	80,960.615	13,144.758^*^	0.85	−40,389.348
4-Profiles	75,935.132	4,977.515^*^	0.85	−3,7851.340
5-Profiles	74,935.000	5,209.913	0.85	−3,5194.834

*BIC, Bayesian information criteria; BLRT, Bootstrapped likelihood ratio test*.

* *p < 0.05*.

**Figure 1:**
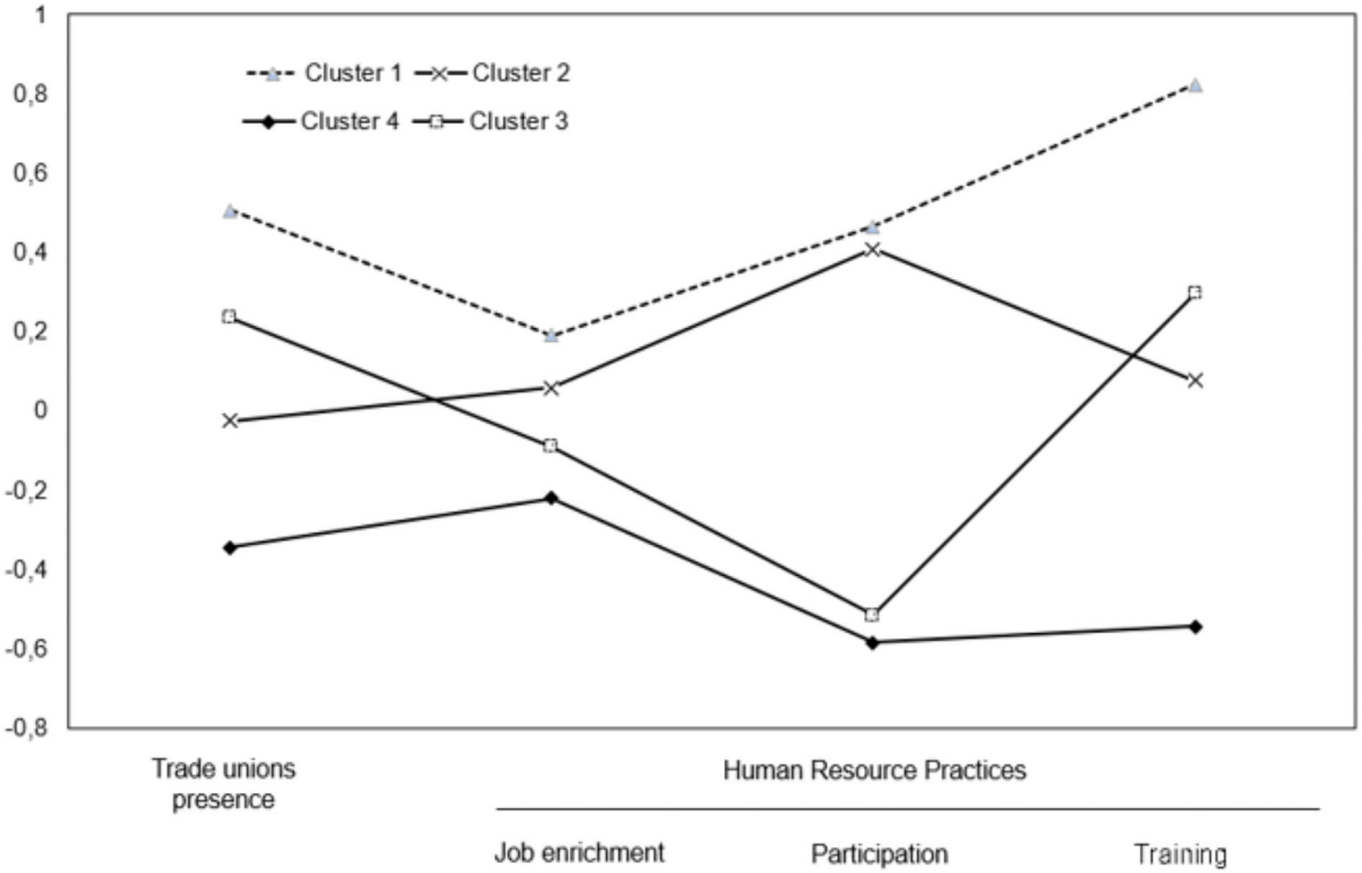
Identified profiles in Europe private organisations.

In Table 3, the posterior probabilities of the four-profile model are shown, values between 0.8 and 1 indicate that the distinction between profiles is well ensured (Nagin, 2005). Therefore, our data support hypothesis 1 where different combinations of union presence and HRPs in the European private sector are predicted. Specifically, four profiles have been identified (see Figure 1).

**Table 3:** Average posterior probabilities for the four-profile model.

**Label**		**1**	**2**	**3**	**4**
Profile 1		**0.95**	0.02	0.00	0.04
Profile 2		0.04	**0.81**	0.03	0.12
Profile 3		0.00	0.02	**0.95**	0.03
Profile 4		0.03	0.05	0.02	**0.90**

*The largest posterior probabilities for each profile are in bold*.

Profile 1 (*n* = 5,821) presented the highest level of HRPs and trade union presence. In this profile, most employees (45.4%) worked in big organisations. Only 16.9% of employees worked in small organisations and the rest (37.7%) in medium-sized organisations. This profile represents employees essentially from the tertiary sector (62%), followed by the secondary (30.8%), the primary (2.9%) sector, and other services activities (4.3%).

Profile 2 (*n* = 8,889) showed high values of HRPs and a low-to-medium value trade union presence. In this profile, most employees (46.7%) worked in small organisations, followed by medium-sized companies (37.3%) and big organisations (16%). There is a higher percentage of employees from the tertiary sector (57.8%) followed by employees from the secondary (29.9%), primary (6.5%), and other service activities (5.8%.) sectors.

Profile 3 (*n* = 2,577) represented high values of trade union presence in opposition to a low value of HRPs, especially participation at work and job enrichment. In this profile, most employees were in medium-sized organisations (46%), and big organisations (37.9%). There is a high percentage of the tertiary sector (59.6%) employees, followed by secondary activities (33.8%), other services activities (4.3%), and the primary sector (2.3%)

Profile 4 (*n* = 7,216) showed very low values in both union presence and HRPs. This profile includes a higher percentage of employees from small organisations (46.9%), or medium-sized organisations (40.5%). Only 12.6% of the employees belonged to big organisations. In this profile, we found the maximum percentage in the primary sector (9%) in comparison to the other profiles. Secondary activities employees represent 26.4%, the tertiary sector represents 59.9%, and other activities constitute 4.7%.

Regarding the number of employees in each profile, the most numerous profiles are profile 2 (*N* = 8,889) and 4 (*N* = 7,216) followed by 1 (*N* = 5,821) and the least numerous being profile 3 (*N* = 2,577). Therefore, it seems that the profiles in which a greater number of people are grouped are those in which union representation is lower. A moderate level of participants are grouped in profile 1, where HRPs are combined with a high union presence; and the least number of people are represented in profile 3, where union presence is a priority.

Considering working contracts, the four profiles follow a similar pattern. The higher percentages are represented by full-time and permanent contract employees (see Table 4). However, if we look across profiles, some differences arise. First, results show how better working contracts, that is, full-time and permanent contracts, are represented in profiles with high presence of HRPs and trade unions or just only high HRPs (profiles 1 and 2, respectively). Second, the higher percentage of employees with part-time, non-permanent contract, ETT, internship, and non-contract is represented in profiles 3 and 4 where the presence of unions is higher than the HRPs or both are under-present, respectively.

**Table 4:** Working contracts in the different profiles.

	**Working hours**		**Contract period**				
	**Part time**	**Full time**	**Permanent**	**Non-permanent**	**TEA**	**Internship**	**No contract**
Profile 1	10.7	86	85.4	8.8	0.9	0.3	3.9
Profile 2	15.4	79.5	84	4.5	0.5	0.2	4.9
Profile 3	19.3	74.9	66.1	16.5	2.1	0.9	11.6
Profile 4	19.4	75.9	71.6	11.1	1.4	0.7	9.5

*All numbers represent percentages*.

### Analyzing profile outcomes. The quality of labor relations and the employees' wellbeing

To test how the different profiles are related to employees' wellbeing and the quality of labor relations, we conducted a variance analysis and *post-hoc* comparisons using SPSS. We used the latent profiles as the grouping variable. Supporting hypothesis 2, the variance analysis showed differences between the patterns in all the indicators of employees' general wellbeing, *F*_(117, 71)_, *p* < 0.001, work engagement, *F*_(655, 45)_, *p* < 0.001, and in the quality of labor relations indicators: perceived justice *F*_(178, 18)_, *p* < 0.001, leadership quality *F*_(446, 80)_, *p* < 0.001, and social support *F*_(541, 22)_, *p* < 0.001. Results for the *post-hoc* analysis comparing the profiles relationship with dependent variables are shown in Table 5.

**Table 5:** Means and significant differences with the four-profile model.

	**Wellbeing**	**Engagement**	**Justice**	**Leadership quality**	**Social support**
Profile 1	2.57	3.09	3.11	3.17	3.14
Profile 2	2.53	3.02	3.11	3.09	3.13
Profile 3	2.31	2.72	2.81	2.70	2.65
Profile 4	2.23	2.61	2.86	2.67	2.62
*Post-hoc*	1 > 2 > 3 > 4^***^	1 > 2 > 3 > 4^***^	1, 2 > 3 > 4^***^	1 > 2 > 3 > 4^***^	1, 2 > 3, 4^***^

*** *p < 0.001*.

Profile 1 – high HRPs/high presence of trade unions – was related to the highest levels of employees' wellbeing. It is also related to higher levels of leadership quality than profile 2 – high HRPs/low-medium presence of trade unions –. In common with profile 2, they show a significant relationship between employees' perceived justice and social support. This corroborates hypothesis 2a since the presence of HRPs and union presence have a positive relationship with employees' wellbeing and the quality of labor. It also corroborates hypothesis 2c, according to which the profile with high levels of HRPs and union presence would obtain the best quality labor relations indicators.

Profile 3 – low HRPs/high presence of trade unions – was significantly outperformed by profiles 1 and 2 in all indicators. Nevertheless, profile 3 had higher levels of employee's wellbeing and quality of labor relations, that is, perceived justice and leadership quality, than profile 4 – low HRPs /low presence of trade unions. Therefore, according to our hypothesis 2b, organisations with lower levels of HRPs and union presence relate to a lower quality of labor relations and employees' wellbeing.

Taken together, and according to our predictions, results show that organisations that combine high levels of HRPs and union presence (profile 1) were related to more successful outcomes (hypothesis 2c), whereas the organisations with lower levels of HRPs and lower levels of union presence (profile 4) were related to less satisfactory outcomes (hypothesis 2b). The remaining two profiles (profile 2 and 3) show intermediate results with respect to profile 1 (hypothesis 2a). To sum up, organisations that combine the trade union presence with HRPs provided better quality of labor relations and employees' wellbeing.

## Discussion

This study analyses how the combination of HRPs and the level of union presence in organisations is related to employees' wellbeing and the quality of labor relations in the European private sector.

We identified four profiles using latent profile analysis according to HRPs and union presence. The results allow us to differentiate four organisational types: Profile 1 - high level of HRPs/high level of union presence-; Profile 2 - high level of HRPs/low-to-medium level of union presence -; Profile 3 - low level of HRPs/high level of union presence-; and Profile 4 - low levels of both.

Related to the organisational sectors and size, profile 1 grouped a majority of big organisations in the service sector. In profile 2, we find medium-sized organisations without a strong union presence but a higher level of HRPs. Profile 3 represents most medium-to-big organisations with a strong union presence. This is the case in factories and manufacturing firms where unions have a very important role and HRPs barely exist. Finally, profile 4 is mainly represented by small organisations where no union presence nor HRPs exist. These results show that the four profiles demonstrated in Guest and Conway's study (1999) are still present. Data also show the existence of a large proportion of organisations that are lacking human resources practices and trade union presence (~30%), which have traditionally been called black hole organisations, and which include a majority of small and medium-sized organisations.

The second research question was to analyse the effects of each of these profiles on employees' wellbeing and the quality of labor relations. The results confirm that profile 1 was related to the best results both in employees' wellbeing and quality of labor relations. This profile is found in big organisations, which are more likely to have a union presence and a large human resources department. In this profile 1, workers perceive greater participation at work and training, being consistent with the “Mutual Gains” theory by Yang et al. (2019), which proposes that unions and HRPs are not exclusive, they mutually enhance each other's influence. It is also in line with Cristiani and Peiro's study (2015, 2018), because unions promote individual-centered human resource practices.

Profile 2 is of interest as we can see the difference between the effect of HRPs. In this profile, we can find a high level of HRPs and a low-to-medium level of unions in medium-sized organisations. Outcomes related to this profile are better than profile 4 (low–low) and profile 3 (low HRPs - medium-to-high union presence). This profile 2 is interesting in comparison to profile 3, as the levels of participation at work are substantially higher in profiles 1 and 2 than in profiles 3 and 4. This participation is not explained by the trade union presence, which is low in profile 2 but is motivated by the human resources practices in the organisation. One explanation of that finding is that in these organisations, a paradigm shift is taking place from collective to individual negotiations, where the employees' new expectations motivate the organisation to comply with the psychological contract through person-centered HRPs (Stirpe et al., 2018).

The comparison between profile 3 and profile 1 suggests that union intervention is not enough without the existence of HRPs in the organisation. Our data contrast with previous studies (e.g., Freeman and Medoff, 1984; Reissner and Pagan, 2013) that have highlighted the importance of the collective voice through formal mechanisms such as the union presence. However, this study demonstrates that the absence of HRPs suggests that union influence is done directly with the employer. This influence is ineffective, as participation at work levels and employees' job enrichment levels are perceived to be low. In this sense, these results support previous research, showing that, in industrial relations systems where more power on the employer side exists, unions only have an impact if their voice is listened to by managers and a constructive relationship exists between them (Wood, 2016). In short, these organisations need to share a common objective between unions and employers to increase the quality of labor relations; and at the same time, the union needs a cooperative context to influence organisational decision making (Ryan and Wallace, 2016).

The worst employees' wellbeing and quality of labor relations are reported by employees in profile 4 (low–low), labeled as the “Black Hole,” representing mainly small companies. In this profile, there is low employee wellbeing and engagement, low leadership quality, low perception of fairness, and low levels of social support. Following Wilkinson et al., 2007 study, they are very traditional organisations in terms of professional management of their employees and very fearful of the effect that a union influence could generate.

Despite the cultural differences in the European context, we find a common paradigm of HRPs and union presence that sheds light on the need for combining both. This study shows how the combination is associated with employee wellbeing and engagement, higher perceptions of fairness and social support, and quality leadership. However, HRPs are not easy to implement and development, especially in small businesses, HRPs require effort and dedication by the organisation. However, their use within all companies independent of their size is of relevance for organisational effectiveness. It would be necessary in this sense that the organisation can rely on people to carry out this function, either external specialists or part-time staff, who could be trained for this purpose. On the other hand, union presence is also necessary to obtain the best results in a work environment increasingly focused on individual negotiations. In this sense, even if the size of the organisation does not legally require a shop steward, our data suggest that the organisation should have people who facilitate communication with workers and who perform the facilitation function that trade unions usually do. Ibsen and Tapia (2017) and Munduate et al. (2013) advocate for the revitalization of unions improving employees' representative competencies. Due to its recent loss of power, a reinvention is necessary, through new content, new political actions, and the construction of alliances with new social movements. To sum up, this research highlights the importance of unions and their alignment with HRPs, since the coexistence of both produces a positive synergy for the employee and the organisation.

### Limitations and future lines of research

The sixth EWCS is designed to be transversal, therefore the possibility of making causal inferences is reduced and there may be common method limitations. However, the large sample used, and its representativeness enhances the value of the design used and it is a proper design to identify profiles using latent profile analysis. Although working with a large and representative sample brings many advantages, some significant relationships between variables with low correlations should be taken with caution. However, in the present study when different HRPs are combined in a cluster, this combination has sufficient potential to differentiate between organisations where employees experience more or less wellbeing. Moreover, the factor analysis performed demonstrates a lack of common method variance (Richardson et al., 2009). However, a future longitudinal study would be beneficial to analyse the nature of the relationship between the identified profiles and the outcome variables. Another limitation relates to the selected HRPs. Other HRPs could be analyzed such as work–life balance practices because these practices are focused on promoting employees' wellbeing and are negotiated in many European countries (Martínez-Corts and Moreno-Beltrán, 2020). This work has focused on the analysis of private sector organisations because it is subject to greater variability in the types of hiring as well as the proliferation of new organisational models. A future study should be extended to the public sector. It would also be interesting to consider these profiles in other different cultural and economic contexts, such as China or Japan. Finally, for our study three specific HRPs are used, namely, development, participation at work, and job enrichment. It would be interesting to extend it to other practices such as retribution or work–family balance practices.

### Practical implications

This study has various practical implications. Understanding the existing relationship between HRPs and union presence is relevant to social agents, union representatives, and organisational leaders. First, HRPs are essential in any organisation, regardless of their size and activity sector. Although the organisational size makes it difficult to have their own human resource department, managers can be advised by external consultants. The challenge is to extend HRPs from large to small organisations. Second, organisations should promote and develop a climate of cooperation with representatives, because when they are aligned, they have a positive impact on employees' wellbeing, and the quality of labor relations improve. For this reason, opposite to a unitary or antagonistic perspective, a synergistic perspective would be the best option.

## Conclusions

The present study shows that organisations that combine HRPs and union presence relate to the highest levels of employee wellbeing and quality of labor relations. However, organisations with a low level of union presence or HRPs reached the worst levels in employees' wellbeing and quality of labor relations indicators.

## Data availability statement

Publicly available datasets were analyzed in this study. This data can be found at: https://www.eurofound.europa.eu/es/data/european-working-conditions-survey.

## Ethics statement

The studies involving human participants were reviewed and approved by Junta de Andalucía, Spain (Code 0254N16). The patients/participants provided their written informed consent to participate in this study.

## Author contributions

All the authors contributed to the conception and design of the work and the acquisition, analysis, and interpretation of data. They drafted the work and revised it critically. The authors gave the final approval of the manuscript before the submission. They participated at every stage of the research process.

## Funding

This publication is part of the I-D-i project (PID2019-110093GB-I00), funded by MCIN/AEI/10.13039/501100011033 [Ministerio de Ciencia e Innovación y Universidades (Spanish Ministry of Science, Innovation and Universities and Spanish Research Agency)].

## Conflict of interest

The authors declare that the research was conducted in the absence of any commercial or financial relationships that could be construed as a potential conflict of interest.

## Publisher's note

All claims expressed in this article are solely those of the authors and do not necessarily represent those of their affiliated organizations, or those of the publisher, the editors and the reviewers. Any product that may be evaluated in this article, or claim that may be made by its manufacturer, is not guaranteed or endorsed by the publisher.
